# Associations between polymorphisms in the myostatin gene with calving difficulty and carcass merit in cattle

**DOI:** 10.1093/jas/skad371

**Published:** 2023-11-03

**Authors:** Cliona A Ryan, Deirdre C Purfield, Saeid Naderi, Donagh P Berry

**Affiliations:** Department of Animal Bioscience, Animal and Grassland Research and Innovation Centre, Teagasc, Moorepark, Fermoy, Co. Cork, Ireland; Department of Biological Sciences, Munster Technological University, Bishopstown, Co. Cork, Ireland; Department of Biological Sciences, Munster Technological University, Bishopstown, Co. Cork, Ireland; Irish Cattle Breeding Federation, Bandon, Co. Cork, Ireland; Department of Animal Bioscience, Animal and Grassland Research and Innovation Centre, Teagasc, Moorepark, Fermoy, Co. Cork, Ireland

**Keywords:** beef cattle, carcass traits, dystocia, GDF8, haplotype, myostatin

## Abstract

A fully functional myostatin gene inhibits muscle fiber growth. The objective of the present study was to quantify the association between 21 known myostatin mutations with both calving and carcass traits in 12 cattle breeds. The myostatin genotypes of 32,770 dam-progeny combinations were used in the association analysis of calving dystocia, with the genotypes of 129,803 animals used in the mixed model association analyses of carcass weight, conformation, and fat score. The mixed model included additive genetic, maternal, and permanent environmental effects where appropriate. The mutant genotypes of nt821, Q204X, and F94L were all associated (*P* < 0.01) with more calving difficulty when present in either the dam or the progeny. The nt821 deletion had the greatest association with calving difficulty when the homozygous deletion was present in either the calf (0.37 points greater calving difficulty score relative to calves carrying no copies of the deletion based on a one to four scale) or the dam (1.30 points greater calving difficulty score relative to dams carrying no copies of the deletion), although the association between the calf’s nt821 genotype and calving difficulty differed depending on the nt821 genotype of the dam. With the exception of nt748_78, nt414, and nt374_51, all other seven segregating myostatin variants were associated (range of allele substitution effect size relative to animals with no copies of the mutant allele) with carcass weight (2.36 kg lighter to 15.56 kg heavier), all 10 segregating variants with conformation (0.15 units less conformed to 2.24 units more conformed assessed on a scale of 1 to 15), and all segregating variants other than E226X with carcass fat (0.23 units less carcass fat cover to 3.85 units more carcass fat cover assessed on a scale of 1 to 15). Of these, the F94L, Q204X, and nt821 mutations generally had the greatest association with all three carcass traits, giving rise to heavier and more conformed carcasses. Despite the antagonistic genetic relationship between calving difficulty and carcass traits, the nt374_51, F94L, and E226X mutations were all associated with improved carcass merit while having minimal expected consequences on calving difficulty. Thus, animals carrying these mutation(s) may have favorable genetic merit for calving difficulty and carcass merit. Furthermore, depending on the dam genotype, a bull with two copies of the nt821 mutation can produce progeny with improved carcass merit while minimizing calving problems.

## Teaser

This study characterized the association between the 21 known myostatin mutations with calving difficulty, carcass fat, carcass conformation, and carcass weight. Pleiotropic variants that were associated with both improved carcass merit and greater calving difficulty were identified, as were variants that were associated with improved carcass merit while having minimal consequences on calving difficulty. The latter enables breeders to select animals with these non-pleiotropic mutations to potentially produce progeny with improved carcass merit while minimizing the risk of calving difficulty.

## Introduction

The discovery of genomic polymorphisms affecting animal performance has many potential downstream applications. For instance, previous studies using both simulations (Teng et al., 2020) and real-life data (Meuwissen and Goddard, 2022) have shown that, in some situations, including known causal polymorphisms in genetic evaluation models can improve the accuracy of the resulting genomic evaluations. The inclusion of causal polymorphisms in the prediction process is likely to be particularly important for mitigating the impact of recombination across generations (including across breeds) on the linkage phase between genotyped markers and the (unknown) causal mutations. Whereas genomic evaluation models simultaneously estimate the effect of the causal mutation(s) and those of all remaining polymorphisms in the same model, knowing the effect of polymorphisms on performance traits is still important for improving selection strategies and designing optimal mating plans. Therefore, an accurate assessment of the impact of causal polymorphisms on all traits of economic importance is valuable.

Many polymorphisms with proven (Grobet et al., 1997) or putative (Chung et al., 2022) effects on performance traits in cattle have been identified. For example, in cattle DGAT1 influences milk production (Grisart et al., 2002), GHR impacts milk yield and composition (Blott et al., 1998), myostatin impacts meat quality (Ménissier, 1982) and SMAD6 influences ovulation rate (Kamalludin et al., 2017). Polymorphisms associated with genetic diseases in cattle have also been identified, such as BLAD (Kehrli et al., 1990), along with mutations resulting in congenital defects such as dwarfism (Yoneda et al., 1999) and osteoporosis (Meyers et al., 2010).

The effects of many of these mutations on animal performance have often been estimated in relatively small populations, and often under limited environmental conditions (Khasanaha et al., 2016; 2021Elzaki et al., 2021). Moreover, the effect (or association for non-causal mutations) of polymorphisms on performance traits with a maternal genetic component (e.g., calving dystocia) are generally estimated considering only the genotype of the animal itself and not its dam (Casas et al., 1999). Widespread genotyping of commercial animals using single nucleotide polymorphism microarray technology has facilitated the creation of large databases of commercial animal-dam pairs called SNP genotypes, often with the genotypes of known causal polymorphisms. These large databases, especially with multiple breeds and crossbreds, also provide an opportunity to investigate the effect of individual polymorphisms conditional on other polymorphisms, especially those which may be in tight linkage disequilibrium (LD) within breed.

The GDF8 (myostatin) gene harbors the allele causing the double-muscle phenotype in cattle (Grobet et al., 1997; McPherron and Lee, 1997), pigs (Qian et al., 2015), sheep (Clop et al., 2006; Boman et al., 2009), and humans (Schuelke et al., 2004). Myostatin acts as a negative regulator for the proliferation of muscle fibers (Grobet et al., 1997; McPherron and Lee, 1997). The myostatin gene in cattle contains 21 known mutations (i.e., SNPs and indels) which are now part of routinely used genotyping panels (Grisolia et al., 2009). The double-muscled syndrome is present in many breeds of cattle albeit often attributable to different mutations in the myostatin gene (Phocas, 2009), which result in partial or complete loss of function of myostatin activity, making it unable to terminate muscle fiber expansion. Previous studies exploring the association between myostatin and performance in cattle have mainly focused on the disruptive (i.e., nt821, Q204X, E226X, E291X, and nt419) or missense mutations (i.e., F94L, and C313Y). However, associations between performance and the other 11 discovered functional myostatin mutations remain to be elucidated (Grisolia et al., 2009). The objective of the present study was to quantify the association between 21 known myostatin mutations with both calving and carcass traits in 12 prominent cattle breeds.

## Materials and Methods

The data used in the present study originated from a preexisting database managed by the Irish Cattle Breeding Federation. Therefore, it was not necessary to obtain animal care and use committee approval in advance of conducting this study. Only myostatin genotypes collected after the EP 2 045 322 B1 patent’s expiration date (September 01, 2018) were used in the current study.

### Genotype data

Genotypes from 50,855 SNPs were available from 1,065,981 dairy and beef cattle generated using a custom Illumina bead chip; the panel included 21 known variants in the myostatin gene (Table 1). All animals and all 50,855 SNPs had a call rate of ≥ 90%. Non-autosomal SNPs and SNPs that did not adhere to Mendelian inheritance patterns were discarded. Following edits, 45,894 SNPs remained which were used to verify purebred status.

**Table 1.** The mutant/wild-type alleles for each of the 21 myostatin variants, the type of mutation, and protein change each mutation causes

Variant	Wild-type/ mutant	Consequence type	HGVS^1^ protein
C313Y	G/A	Missense	p.Cyc313Tyr
D182N	G/A	Missense	p.Asp182Asn
E226X	G/T	Nonsense	p.Glu226X
E291X	G/T	Nonsense	p.Glu291X
F140L	TTAAATT/−	Nonsense	p.Phe140LeufsX10
F94L	C/A	Missense	p.Phe94Leu
L64P	T/C	Missense	p.Leu64Pro
nt267	A/G	Silent	—
nt324	C/T	Silent	D
nt374_16	T/−	Silent	—
nt374_50	G/A	Silent	—
nt374_51	C/T	Silent	—
nt387	G/A	Silent	T
nt414	C/T	Silent	C
nt419	TTAAATT/AAGCATACAA	Nonsense	p.Phe140X
nt747 + 11	A/G	Silent	—
nt747 + 7	G/A	Silent	—
nt748_78	T/−	Silent	—
nt821	ATGAACACTCC/−	Nonsense	p.Glu275ArgfsX14
Q204X	C/T	Nonsense	p.Gln204X
S105C	C/G	Missense	p.Ser105Cys

^1^HGVS (Human Genome Variation Society).

#### Establishment of purebred and crossbred populations

**Figure 1.**
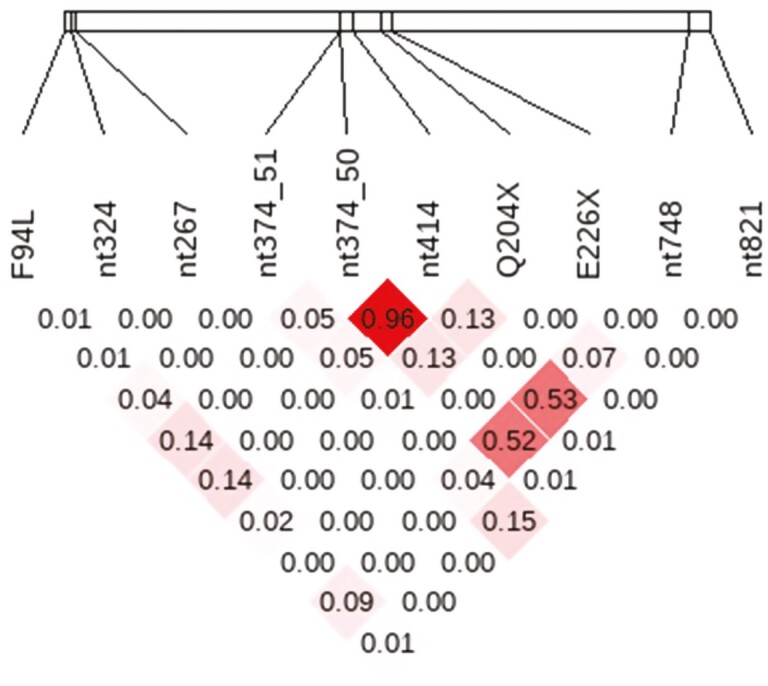
The *r*^2^ linkage disequilibrium between all 10 segregating myostatin variants in the admixed population.

In order to determine the percentage of animals in each breed carrying at least one mutant allele for each myostatin variant, purebred breed-specific populations were established using the available genotype information coupled with the methods described by Ryan et al. (2023) to estimate breed composition of individual animals. In brief, an unsupervised analysis using Admixture software (Alexander et al., 2009) was applied to determine the breed composition of all genotyped animals from 12 common Irish beef and dairy breeds. From this, 137,147 individuals with an estimated breed composition of ≥90% to a single breed were considered purebred for that breed (Table 2). A principal component analysis (PCA) was performed on these animals to verify that all putative purebred animals resided within their respective breed cluster in the PCA plot. In addition, a known admixed population was also established for the subsequent association analysis. This crossbred population was defined as genotyped animals with < 90% of its breed composition attributed to one breed in the Admixture analysis; this population consisted of 926,228 genotyped animals. The *r*^2^ LD between the segregating myostatin variants was calculated using Haploview (Barrett et al., 2004) within each breed separately based on the established purebred population (Supplementary Figure S1) and across the admixed population used in the association analyses (Figure 1).

**Table 2.** Number of the purebred genotyped animals per breed

Breed	Number
Angus	26,396
Aubrac	3,455
Blonde d’aquitaine	782
Belgian blue	396
Charolais	21,062
Friesian	228
Hereford	15,172
Holstein	12,532
Limousin	37,661
Salers	3,539
Shorthorn	4,577
Simmental	11,347
Total number	137,147

#### Genotypes and haplotype construction

The genotypes of each of the 21 known mutant myostatin variants were available for each animal in the population (Table 1). The mutant allele for each variant was denoted by mh, while the wild-type allele was represented as +; animals carrying two copies of the mutant allele had a genotype denoted as mh/mh, the homozygous wild-type genotype was +/+ and the heterozygous genotype was indicated by mh/+. The 21 myostatin variants were phased using the entire population of 1,065,981 purebred and crossbred animals in Eagle2 V2.4.1 (Loh et al., 2016); from this, a paternal and maternal haplotype was formed for each animal.

The percentage of animals carrying at least one mutant allele for each of the 21 variants and the frequency of each haplotype per breed was estimated within each of the 12 breeds of the purebred-verified population. For the haplotype association analysis, only haplotypes with a frequency of ≥1% within breed were investigated; a total of 22 haplotypes had a frequency ≥1% while 130 had a frequency <1%. An across-breed haplotype frequency was calculated as the unweighted average of the within-breed haplotype frequencies to avoid bias due to different population sizes in each of the 12 breeds.

### Phenotypes

#### Calving difficulty

Calving information was available for 17,338,536 calving events from Irish beef and dairy cows between the years 2004 and 2021, inclusive. Calving difficulty in Ireland is scored by producers on a four-point scale as: (1) no assistance, (2) some assistance (assistance by one person), (3) considerable assistance (assistance by more than one person or assistance with a calf puller), and (4) veterinary assistance (including cesarean). A total of 44,969 calving records where cow parity was >15 were discarded, as were the records from cows that calved more than 425 d before or after the respective ­parity median. Furthermore, first-parity cows recorded to have calved younger than 660 d of age were discarded. Records from twin births and embryo transfers were also omitted. Additionally, 4,235,289 calving records from herd-years which had recorded no variability in calving difficulty scores were removed, as were 8,236,797 calving records from herd-years with <5 genotyped dam-progeny combinations. Further to this, a total of 184,843 animals without a recorded sire were omitted, as were 917,224 animals who were registered in a breed society. Following edits, calving difficulty data remained on 2,780,435 animals from 1,352,397 dams; within this population, there were 59,615 pairs of dams and progeny where both individuals were genotyped.

Contemporary group was defined as herd-year-season of calving using an algorithm described in detail by Schmitz et al. (1991) and Crump et al. (1997) and used in the Irish national genetic evaluations. The algorithm initially grouped animals, which calved within 10 d of each other together within a given herd. Where < 10 animals were clustered together, they were grouped with an adjacent contemporary group in the same herd, but only where the number of days between the start of the earliest contemporary group and the end of the latest contemporary group was <90 d. Contemporary groups with <3 records were discarded, resulting in 32,770 genotyped dam-progeny pairs in 3,092 genotyped contemporary groups. All contemporary groups had variability in ­calving difficulty scores.

#### Carcass traits

The three carcass phenotypes investigated were carcass weight, carcass conformation, and carcass fat. Carcass weight is measured, on average, 1 h after slaughter, ­following the removal of the head, hide, legs, thoracic and abdominal organs, and internal fat. Carcass conformation reflects the shape and development of the carcass, while carcass fat reflects the level of fat cover on the outside of the carcass as well as within the thoracic cavity (Kenny et al., 2020). Carcass conformation and fat scores were graded under the European Union beef carcass classification system (EUROP), as described by Pabiou et al. (2012). Under the EUROP grading system, carcass conformation scores are represented by the letter E (best), U, R, O, and P (worst), which are subdivided into three sub-scores (i.e., −, = and +). Carcass fat scores are represented by the numbers 1 (lowest fat cover), 2, 3, 4, and 5 (highest fat cover), with the same three sub-scores applied (i.e., −, = and +). As described by Englishby et al. (2016), the resulting EUROP classification grades for carcass fat and conformation were translated into a 1 to 15 scale, where a score of 1 relates to poor conformation and low-fat cover, while that of 15 relates to excellent conformation and high-fat cover.

A general heterosis coefficient and recombination loss coefficient were calculated for all animals in the dataset using the formulae outlined by VanRaden and Sanders (2003):


$$\begin{array}{l} Heterosis=1-\sum_{i=1}^{n}sire_{i\;}\times dam_{i}\; \end{array}$$

$$\begin{array}{l} Recombination\;\;\;loss=1-\sum_{i=1}^{n}\frac{{sire}_{i}^{2}\times \;{dam}_{i}^{2}}{2} \end{array}$$

where sire_*i*_ and dam_*i*_ are the proportion of breed *i* in the breed composition of the sire and dam, respectively.

Only steers and heifers slaughtered between the ages of 14 and 36 mo, inclusive, and young bulls slaughtered between 12 and 24 mo, with recorded carcass weights between 100 and 800 kg were considered. Furthermore, any steer, heifer, or young bull born to a dam in parity > 10, or from embryo transfer were omitted. Carcass records from cattle that resided in more than two herds during their lifetime or moved herds within 100 d prior to slaughter were not considered further. Additionally, animals registered with a breed society were also omitted from the carcass analyses. Following all edits, carcass data remained for 137,198 genotyped animals. These animals were allocated to herd-year-sex contemporary group using an algorithm described by Berry and Evans (2014) to group animals of the same sex (i.e., heifers, steers, and young bulls) that were slaughtered from the same herd within 60 d of one another, to account for management differences among herds. Only animals in contemporary groups containing at least five genotyped animals were retained. Following edits, 129,803 animals remained. Of these, 2,914 animals were also included in the dystocia analyses.

### Association analyses

#### Calving difficulty

The association between segregating myostatin variants and calving difficulty, as well as the association between myostatin haplotypes and calving difficulty, were determined separately using a series of animal-dam linear mixed models in the ASreml software suite (Gilmour et al., 2015). Both series of analyses followed a similar methodology; in the first series of analyses, the number of mutant alleles for individual myostatin variant(s) of both the calf and the dam were included as fixed effects. In the second series of analyses, the number of copies of the derived myostatin haplotypes of the calf and the dam were included in the model as separate fixed effects. Both purebred and crossbred dam-progeny combinations were included in both series of analyses. The statistical model used was


$$\begin{array}{l} \begin{array}{llll} & y=sex+het_{a}+het_{d}+rec_{a}+rec_{d}+parity|age+parity+\; & \;\sum_{n=1}^{k}breed_{ka}+\sum_{n=1}^{k}breed_{kd}\;+MSTN_{a}+MSTN_{d}+CG+\; & \;Calf_{a}+Dam_{m}+Dam_{pe}+e \end{array} \end{array}$$ (1)

where *y* was the dependant variable of calving difficulty; sex was the fixed effect of the sex of the calf; het_a_ was the fixed effect of a general heterosis coefficient of the calf; het_d_ was the fixed effect of a general heterosis coefficient of the dam; rec_a_ was the fixed effect of a general recombination loss coefficient of the calf; rec_d_ was the fixed effect of a general recombination loss coefficient of the dam, parity|age was the fixed effect of the interaction between dam parity and age at calving relative to the parity median; breed_ka_ was the fixed effect of the calf’s breed proportion for each of the 12 breeds; breed_kd_ was the fixed effect of the dam’s breed proportion for each of the 12 breeds; MSTN_a_ was the class effect of either the number of copies of the mutant allele for the myostatin variant(s) under investigation, or the number of copies of the haplotype under investigation carried by the calf (i.e., 0, 1, or 2); MSTN_d_ was the class effect of either the number of copies of the mutant allele for the myostatin variant(s) under investigation, or the number of copies of the haplotype under investigation carried by the dam (i.e., 0, 1, or 2); CG was the random effect for contemporary group where CG ~ *N* (0, $I\sigma_{CG}^{2}$) with $\sigma_{CG}^{2}$ denoting the contemporary group variance and ***I*** denoting the identity matrix; Calf_a_ was the direct polygenic effect of each calf which was assumed to have the distribution *N*(0, $A\sigma_{g}^{2}$), where ***A*** was the numerator ­relationship matrix and $\sigma_{g}^{2}$ was the direct additive genetic variance; Dam_m_ was the maternal genetic effect which had the distribution *N*(0, $A\sigma_{m}^{2}$) where $\sigma_{m}^{2}$ was the maternal genetic variance; Dam_pe_ was the permanent environmental effect due to the dam which had the distribution *N*(0, $I\sigma_{pe}^{2}$) where $\sigma_{pe}^{2}$ was the maternal permanent environmental variation and e was the random residual factor, where *e* ~ *N* (0, $I\sigma_{e}^{2}$) with $\sigma_{e}^{2}$ representing the residual variance.

Initially, each myostatin variant or haplotype was tested individually in the model, with both the dam and the calf genotype for that variant included concurrently, or with the calf and dam haplotype included concurrently. An *F*-test was used to determine the association between each myostatin variant or haplotype and calving difficulty. Where a given myostatin variant or haplotype of the calf was associated (*P* < 0.05) with calving difficulty, a supplementary series of analyses were conducted to determine if the association between the calf’s myostatin genotype and calving difficulty was dependent on the myostatin genotype of the dam (and vice versa). Therefore, for these analyses, a fixed class effect representing an interaction term modeled by concatenating the genotype/haplotype of the calf with that of the dam was also included in the model previously described. Finally, all variants associated with dystocia (*P* < 0.05) when carried by the dam or progeny were included in the model concurrently to test for associations independent of other variants.

#### Carcass traits

The association between segregating myostatin variants or myostatin haplotypes with carcass weight, conformation, and fat was determined using a series of animal linear mixed models in ASreml (Gilmour et al., 2015). Both purebred and crossbred animals were included in the analysis. The statistical model used was


$$\begin{array}{lll} y= & +het_{a}+rec_{a}+parity+age|sex+CG\; & +\sum_{n=1}^{k}breed_{ka}+MSTN_{a}+Animal_{a}+e \end{array}$$ (2)

where *y* was the recorded carcass phenotype of the animal; het_*a*_, rec_*a*_, parity, $\sum_{n=1}^{k}Breed_{ka}$, MSTN_*a*_, Animal_*a*_, and *e* are as previously described in equation (1); age|sex was the fixed effect of the interaction between age in months at slaughter and the sex of the animal, i.e., heifer, steer, or young bull; CG was the fixed effect for contemporary group. Initially, each myostatin variant and haplotype was tested individually in the model before all variants associated with the carcass phenotype under investigation (*P* < 0.05) were included in the model concurrently.

## Results

### Allele frequencies

Of the 21 myostatin variants investigated, 11 did not segregate in any of the 12 purebred sample populations (Table 2). The percent of animals carrying at least one mutant allele per breed for all 10 remaining segregating variants is shown in Table 3. Of the 37,661 purebred Limousins, 99% carried at least one copy of the F94L mutation; 77% and 22% were homozygous mh/mh and heterozygous mh/+ carriers for the mutant allele, respectively (Table 3). Similarly, 99% of the purebred Belgian Blue population had two copies of the nt821 deletion. The Q204X variant segregated in four of the purebred populations investigated, but only the Charolais and Limousin populations had more than 1% of their animals carrying at least one copy of the mutant allele. The E226X variant was segregating solely in the Shorthorn population. The variants nt374_50, nt374_51, nt414, and nt748 were segregating in almost all of the breeds investigated (Table 3).

**Table 3.** The percent of purebred^1^ animals carrying at least one mutant allele for each of the segregating myostatin variants

Variant	AA	AU	BA	BB	CH	FR	HE	HO	LM	SA	SH	SI	Mean across breed frequency
E226X	0	0	0	0	0	0	0	0	0	0	14	0	1
F94L	1	98	8	0	27	0	0	0	99	1	1	1	20
nt267	1	0	2	0	0	0	0	0	0	20	0	28	4
nt324	0	7	0	0	8	0	0	0	0	0	0	7	2
nt374_50	38	6	0	0	53	63	18	69	15	65	25	49	33
nt374_51	22	64	60	100	18	9	7	3	53	19	28	3	32
nt414	37	5	0	0	53	63	84	69	13	65	25	49	39
nt748_78	40	7	2	3	56	66	84	72	16	66	27	50	41
nt821	6	9	0	99	0	0	0	0	6	3	7	0	11
Q204X	0	1	0	0	27	0	0	0	8	0	0	1	3

^1^Breeds include Angus (AA), Aubrac (AU), Blonde d’Aquitaine (BA), Belgian Blue (BB), Charolais (CH), Friesian (FR), Hereford (HE), Holstein (HO), Limousin (LM), Salers (SA), Shorthorn (SH), and Simmental (SI).

### Haplotype construction

While 152 distinct haplotypes were detected across all breeds, only 22 had a frequency of ≥1% in any of the purebred populations. These 22 haplotypes accounted for 97% of the haplotypes detected in the entire purebred population, with the most prevalent haplotype occurring at least once in 43% of all purebred animals (Table 4). This haplotype was a wild-type haplotype, as it had the wild-type allele for all of the 10 segregating variants (Table 4). Despite the high frequency of this wild-type haplotype across all breeds, it was not detected in the Belgian Blue population, and only 10% of the Limousin population were carriers of the wild-type haplotype (Table 5). The haplotype containing the mutant allele for all of nt748_78, nt414, and nt374_50 variants was the second most prevalent haplotype, with 19% of the entire purebred population carrying at least one copy of this haplotype (Table 4). This haplotype was detected in 8 of the 12 breeds investigated (Table 5), with the strongest LD occurring within the admixed population between the three mutant alleles in this haplotype (Figure 1). Of the 22 haplotypes with a ­within-breed frequency of > 1%, 8 were breed-specific haplotypes. These breed-specific haplotypes existed in the Charolais, Limousin, Shorthorn, and Salers populations (Table 5) and ranged in frequency from 1.13% (Limousin) to 9.8% (Charolais). Only 6 of the 22 haplotypes were common across two or more breeds (Table 5).

**Table 4.** The alleles of each of the 22 frequent haplotypes detected

	nt748_78	F94L	nt324	nt267	nt374_51	nt374_50	nt414	Q204X	E226X	nt821
Mutant allele	—	A	T	G	T	A	T	T	T	—
Haplotype 1	T	C	C	A	C	G	C	C	G	ATGAACACTCC
Haplotype 2	—	C	C	A	C	A	T	C	G	ATGAACACTCC
Haplotype 3	T	A	C	A	C	G	C	C	G	ATGAACACTCC
Haplotype 4	T	C	C	A	T	G	C	C	G	—
Haplotype 5	T	A	C	A	T	G	C	C	G	ATGAACACTCC
Haplotype 6	T	C	C	A	T	G	C	C	G	ATGAACACTCC
Haplotype 7	—	C	C	A	C	G	C	C	G	ATGAACACTCC
Haplotype 8	T	C	C	G	C	G	C	C	G	ATGAACACTCC
Haplotype 9	—	C	C	A	C	A	T	T	G	ATGAACACTCC
Haplotype 10	—	C	C	A	T	A	T	C	G	ATGAACACTCC
Haplotype 11	—	C	C	G	C	A	T	C	G	ATGAACACTCC
Haplotype 12	T	A	C	A	T	G	C	C	G	—
Haplotype 13	T	C	C	A	T	G	C	C	T	ATGAACACTCC
Haplotype 14	T	C	C	A	C	A	T	C	G	ATGAACACTCC
Haplotype 15	—	A	C	A	C	A	T	T	G	ATGAACACTCC
Haplotype 16	—	A	C	A	C	A	T	C	G	ATGAACACTCC
Haplotype 17	T	C	T	A	C	G	C	C	G	ATGAACACTCC
Haplotype 18	—	C	C	A	T	G	C	C	G	—
Haplotype 19	T	A	T	A	T	G	C	C	G	ATGAACACTCC
Haplotype 20	T	C	C	G	T	G	C	C	G	ATGAACACTCC
Haplotype 21	—	A	C	A	C	G	C	C	G	ATGAACACTCC
Haplotype 22	—	A	C	A	T	A	T	T	G	ATGAACACTCC

**Table 5.** The percentage frequency of each haplotype within each breed^1^, as well as separately across the entire purebred population

Haplotype	AA	AU	BA	BB	CH	FR	HE	HO	LM	SA	SH	SI	Across breed % haplotype frequency^2^
Haplotype 1	66.4	11.2	59.9	0	52.4	54.8	36.1	51.4	10.1	45.7	71.1	56.2	42.9
Haplotype 2	17.3	0	0	0	12.1	35.3	55.0	42.3	0	31.9	10.6	21.1	18.8
Haplotype 3	0	44.2	1.9	0	6.7	0	0	0	51.1	0	0	0	8.6
Haplotype 4	2.3	0	0	97.7	0	0	0	0	0	0	2.6	0	8.5
Haplotype 5	0	30.3	1.4	0	2.0	0	0	0	25.4	0	0	0	4.9
Haplotype 6	6.6	2.7	33.6	0	3.1	3.0	1.0	0	1.1	3.3	4.1	0	4.9
Haplotype 7	2.3	0	0	0	1.0	3.7	1.3	3.1	0	1.6	1.6	1.4	1.3
Haplotype 8	0	0	0	0	0	0	0	0	0	4.0	0	9.4	1.1
Haplotype 9	0	0	0	0	9.8	0	0	0	0	0	0	0	0.8
Haplotype 10	1.6	0	0	0	1.0	1.5	2.3	0	0	2.7	0	0	0.7
Haplotype 11	0	0	0	0	0	0	0	0	0	3.3	0	4.3	0.6
Haplotype 12	0	3.8	0	0	0	0	0	0	2.0	0	0	0	0.5
Haplotype 13	0	0	0	0	0	0	0	0	0	0	5.7	0	0.4
Haplotype 14	0	0	0	0	0	1.3	2.4	0	0	1.3	0	0	0.4
Haplotype 15	0	0	0	0	1.9	0	0	0	2.4	0	0	0	0.3
Haplotype 16	0	0	0	0	2.0	0	0	0	1.5	0	0	0	0.3
Haplotype 17	0	0	0	0	1.3	0	0	0	0	0	0	1.6	0.2
Haplotype 18	0	0	0	2.0	0	0	0	0	0	0	0	0	0.1
Haplotype 19	0	1.8	0	0	0	0	0	0	0	0	0	0	0.1
Haplotype 20	0	0	0	0	0	0	0	0	0	1.6	0	0	0.1
Haplotype 21	0	0	0	0	0	0	0	0	1.1	0	0	0	0.1
Haplotype 22	0	0	0	0	0	0	0	0	1.1	0	0	0	0.0

^1^Angus (AA), Aubrac (AU), Blonde d’Aquitaine (BA), Belgian Blue (BB), Charolais (CH), Friesian (FR), Hereford (HE), Holstein (HO), Limousin (LM), Salers (SA), Shorthorn (SH), and Simmental (SI).

^2^The across-breed haplotype frequency was calculated as the unweighted average of the haplotype frequencies calculated in each breed individually to avoid bias due to different population sizes in each of the 12 breeds.

### Calving difficulty

More than 90% of the 32,770 recorded calvings required no assistance at birth, 7.5% required some assistance, 1.3% required considerable assistance, and just 0.3% (109 calving events in total) required veterinary assistance. The observed effect on mean calving difficulty for heterozygous and homozygous mutant genotypes for the 10 segregating myostatin variants relative to the homozygous wild-type genotype is in Table 6. Of the 10 segregating variants, the mutant genotypes of nt821, Q204X, and F94L were all associated (*P* < 0.001) with calving difficulty when present in either the dam or the progeny. Additionally, the nt374_51 and nt324 mutant genotypes were also associated (*P* < 0.05) with increased calving difficulty, but only when these mutations were present in the calf. The nt821 deletion had the greatest observed impact on calving difficulty when present in either the progeny or the dam (Table 6). A calf that was homozygous mutant for nt821 (mh/mh) had, on average, a calving difficulty score 0.37 units higher than a calf that was homozygous wild type (+/+) even after adjusting for nuisance factors in the model (including the dam nt821 variant). Similarly, dams that were homozygous for the nt821 deletion had calving difficulty scores that were on average 1.30 units higher (i.e., worse) than homozygous wild-type dams (+/+).

**Table 6.** The effect^1^ (standard error in parentheses) of the calf or dam mutant allele (mh) relative to the wild-type genotype (+/+) on calving difficulty for each myostatin variant with no interaction considered in the model

		Calf		Dam	
Variant	Genotype	Effect(SE)	*P*-value	Effect(SE)	*P*-value
E226X	mh/+	0.11(0.07)	0.1	−0.06(0.08)	0.47
E226X	mh/mh	NE	NE	NE	NE
F94L	mh/+	0.03(0.01)	0.01	−0.02(0.01)	0.01
F94L	mh/mh	0.08(0.02)	4 × 10^−5^	−0.01(0.02)	0.45
nt267	mh/+	−1 × 10^−3^(0.01)	0.91	0.01(0.01)	0.46
nt267	mh/mh	−0.10(0.08)	0.22	−0.04(0.09)	0.58
nt324	mh/+	−0.07(0.03)	0.02	0.03(0.03)	0.43
nt324	mh/mh	NE	NE	−0.03(0.25)	0.89
nt374_50	mh/+	−8 × 10^−4^(9 × 10^−3^)	0.87	0.01(4 × 10^−3^)	0.29
nt374_50	mh/mh	3 × 10^−4^(5 × 10^−3^)	0.97	1 × 10^−3^(9 × 10^−3^)	0.87
nt374_51	mh/+	0.01(7 × 10^−3^)	0.11	−1 × 10^−3^(7 × 10^−3^)	0.98
nt374_51	mh/mh	0.05(0.02)	0.03	−0.03(0.03)	0.19
nt414	mh/+	−8 × 10^−4^(5 × 10^−3^)	0.86	5 × 10^−3^(4 × 10^−3^)	0.24
nt414	mh/mh	−1 × 10^−3^(9 × 10^−3^)	0.91	−2 × 10^−4^(9 × 10^−3^)	0.98
nt748_78	mh/+	1 × 10^−3^(0.01)	0.8	2 × 10^−3^(4 × 10^−3^)	0.58
nt748_78	mh/mh	4 × 10^−3^(0.01)	0.62	−4 × 10^−3^(0.01)	0.66
nt821	mh/+	0.13(0.02)	1 × 10^−10^	0.05(0.01)	2 × 10^−3^
nt821	mh/mh	0.37(0.11)	8 × 10^−4^	1.30(0.13)	6 × 10^−23^
Q204X	mh/+	0.08(0.01)	1 × 10^−5^	−0.06(0.02)	0.02
Q204X	mh/mh	0.23(0.15)	0.12	NE	NE

^1^NE (non-estimable) refers to genotypes not present in the dataset.

**Figure 2.**
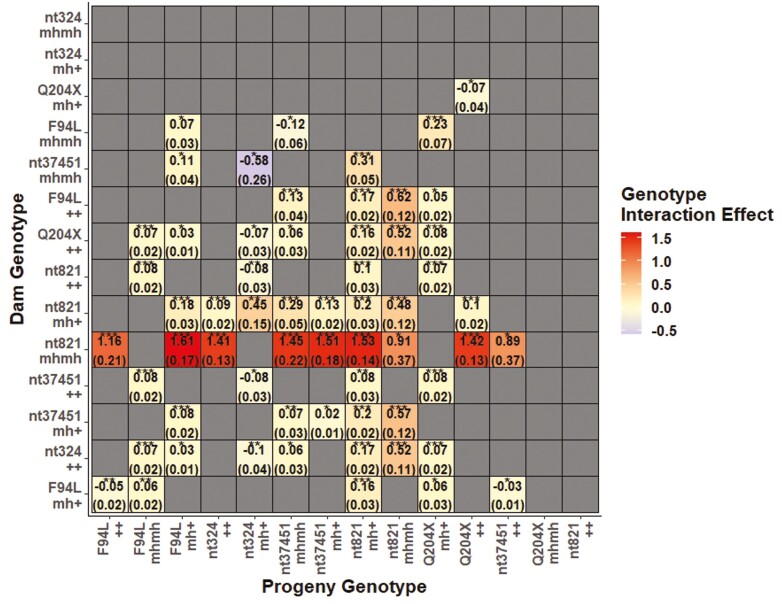
The interaction effect (*P* < 0.05) for each dam-progeny genotype combination, with ‘+’ denoting the wild-type allele and ‘mh’ denoting the mutant allele. The standard error of the estimates is shown in parentheses. Blank squares indicate either the absence of a significant interaction effect or that the dam-progeny genotype combination was not present in the data. A *P*-value of < 0.05, 0.01, and 0.001 is illustrated by *, **, and ***, respectively.

For some of the myostatin variants, the association between the calf’s genotype and calving difficulty differed depending on the genotype of the dam (and vice versa). While both the dam and calf genotypes independently contributed to calving difficulty, the combined genotype effects of some of the dam-progeny genotype combinations were greater than (or less than) what would be expected simply from the sum of their individual genotype effects alone. The difference between joint and marginal genotype dependence is shown in Figure 2. For example, the combined genotype effects of dam-progeny combinations homozygous for the nt821 deletion was 0.91 calving scores higher than the sum of their genotype effects where no interaction was considered.

If all the variants associated with calving difficulty (*P* < 0.05) when carried by the progeny or dam were fitted in a model together, nt374_51 and nt324 were no longer associated with calving difficulty. nt821, Q204X, and F94L remained associated with calving difficulty and had similar allele substitution effects to the single locus association analysis (Table 8).

**Figure 3.**
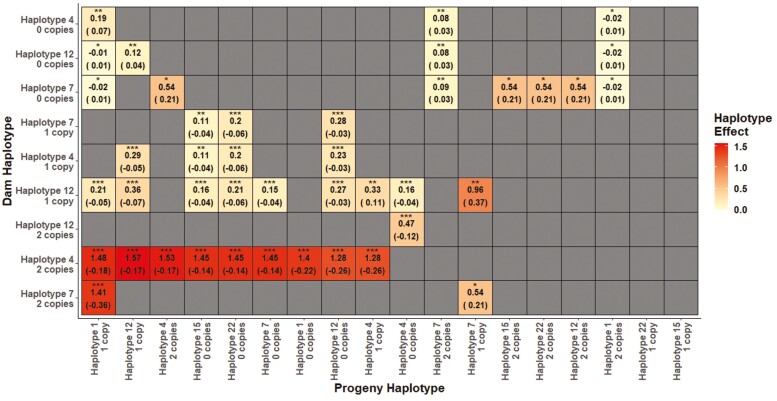
The interaction effect (*P* < 0.05) for each dam-progeny haplotype^1^ combination, comparing if 0, 1, or 2 copies of the haplotype were carried by the calf and dam. The standard error is in parenthesis. Blank squares indicate either the absence of a significant interaction effect or that the dam-progeny haplotype combination was not present in the data. A *P*-value of < 0.05, 0.01, and 0.001 is illustrated by *, **, and ***, respectively. ^1^ Haplotype 1—no mutations; Haplotype 7 – nt748_78 mutation; Haplotype 15 - F94L, nt374_50, nt414, Q204X mutations; Haplotype 22 - nt748_78, F94L, nt374_50, nt374_51, nt414, Q204X, E226X mutations; Haplotype 12 - F94L, nt374_51, nt821 mutations; Haplotype 4 – nt821, nt748_78 mutations.

Only 7 of the 22 haplotypes were associated with dystocia when carried by the progeny (*P* < 0.05), with just four haplotypes (3 of which were a subset of the 7) associated with dystocia when carried by the dam (Table 7). The haplotype containing just the mutant alleles for both nt821 and nt374_51 (haplotype 4; Table 4) had the largest observed association with calving difficulty relative to the wild-type haplotype (Table 4) when carried by both the calf (0.35 points) and the dam (1.4 points), even after adjusting for nuisance factors in the model. For some haplotypes, the association between the calf haplotype and calving difficulty differed depending on the haplotype carried by the dam, most notably when the calf carried one copy of the haplotype containing just the F94L, nt374_51, and nt821 mutations, and the dam carried two copies of the haplotype containing just the nt821 and nt374_51 mutations. In this instance, the combined haplotype effects of the dam-progeny combination were 1.0 calving difficulty score higher than what would be expected from the sum of their individual haplotype effects alone. The difference between joint and marginal haplotype dependence is shown in Figure 3.

**Table 7.** The number of copies of each haplotype carried by the calf and dam, the mutant alleles contained in each haplotype, and the effect^1^ (standard error in parentheses) of carrying 1 or 2 copies (vs. no copy) of that haplotype on calving difficultly

			Calf		Dam	
Haplotype	Mutant alleles	Number of copies	Effect(SE)	*P*-value	Effect(SE)	*P*-value
1	None	1	−0.01(0.01)	0.03	2 × 10^−3^(7 × 10^−3^)	0.84
1	None	2	−0.02(0.01)	0.03	2 × 10^−3^(8 × 10^−3^)	0.85
2	nt748_78, nt374_50, nt414	1	−1 × 10^−3^(4 × 10^−3^)	0.81	2 × 10^−3^(5 × 10^−3^)	0.76
2	nt748_78, nt374_50, nt414	2	−1 × 10^−3^(0.01)	0.89	1 × 10^−3^(9 × 10^−3^)	0.91
3	F94L	1	−0.01(0.01)	0.21	−3 × 10^3^(9 × 10^−3^)	0.81
3	F94L	2	0.02(0.02)	0.34	0.01(0.02)	0.60
4	nt821, nt374_51	1	0.02(0.02)	0.33	0.01(0.02)	0.62
4	nt821, nt374_51	2	0.35(0.11)	2 × 10^−3^	1.45(0.14)	4 × 10^−25^
5	F94L, nt374_51	1	0.01(0.01)	0.50	−0.01(0.02)	0.42
5	F94L, nt374_51	2	−0.02(0.05)	0.60	0.02(0.07)	0.75
6	nt374_51	1	2 × 10^−3^(0.01)	0.84	−1 × 10^−3^(0.01)	0.88
6	nt374_51	2	−0.08(0.08)	0.32	−0.08(0.07)	0.24
7	nt748_78	1	−2 × 10^−3^(0.02)	0.87	−0.02(0.02)	0.23
7	nt748_78	2	0.08(0.03)	4 × 10^−3^	0.54(0.21)	0.01
8	nt267	1	−2 × 10^−4^(0.02)	0.99	−0.02(0.02)	0.44
8	nt267	2	−0.15(0.13)	0.25	−0.17(0.13)	0.19
9	Q204X,nt414, nt374_50,nt748_78	1	0.03(0.02)	0.17	−0.06(0.03)	0.09
9	Q204X,nt414, nt374_50,nt748_78	2	0.21(0.15)	0.17	NE	NE
10	nt414, nt374_50,nt374_51,nt748_78	1	−0.01(0.02)	0.54	−9 × 10^−4^(0.02)	0.96
10	nt414, nt374_50,nt374_51,nt748_78	2	NE	NE	NE	NE
11	nt414, nt374_50,nt748_78, nt267	1	−3 × 10^−3^(0.02)	0.85	0.03(0.02)	0.13
11	nt414, nt374_50,nt748_78, nt267	2	−0.06(0.36)	0.88	−0.03(0.25)	0.89
12	F94L, nt374_51, nt821	1	0.27(0.03)	1 × 10^−22^	0.11(0.04)	3 × 10^−3^
12	F94L, nt374_51, nt821	2	NE	NE	NE	NE
13	E226X, nt374_51	1	0.13(0.10)	0.19	−0.26(0.19)	0.17
13	E226X, nt374_51	2	NE	NE	NE	NE
14	nt414, nt374_50	1	0.04(0.03)	0.17	−0.04(0.04)	0.32
14	nt414, nt374_50	2	−0.01(0.04)	0.86	NE	NE
15	F94L,nt374_50,nt414,Q204X	1	0.11(0.04)	2 × 10^−3^	0.03(0.06)	0.54
15	F94L,nt374_50,nt414,Q204X	2	NE	NE	NE	NE
16	F94L,nt374_50,nt414	1	−0.01(0.01)	0.34	−0.01(0.01)	0.55
16	F94L,nt374_50,nt414	2	NE	NE	NE	NE
17	nt324	1	−0.03(0.05)	0.49	−0.01(0.05)	0.89
17	nt324	2	NE	NE	NE	NE
18	nt748_78, nt374_51, nt821	1	0.13(0.09)	0.15	−0.3(0.12)	0.01
18	nt748_78, nt374_51, nt821	2	NE	NE	NE	NE
19	F94L, nt324, nt374_51	1	0.03(0.08)	0.69	−0.16(0.11)	0.17
19	F94L, nt324, nt374_51	2	NE	NE	NE	NE
20	nt267, nt374_51	1	−0.07(0.06)	0.23	0.09(0.10)	0.36
20	nt267, nt374_51	2	NE	NE	NE	NE
21	nt748_78, F94L	1	−0.04(0.05)	0.44	−0.03(0.05)	0.55
21	nt748_78, F94L	2	−0.05(0.08)	0.52	NE	NE
22	nt748_78,F94L,nt374_50,nt374_51,nt414,Q204X,E226X	1	0.20(0.06)	6 × 10^−4^	−0.11(0.12)	0.36
22	nt748_78,F94L,nt374_50,nt374_51,nt414,Q204X,E226X	2	NE	NE	NE	NE

^1^NE refers to when no animals were present in this study with that genotype.

### Carcass traits

**Figure 4.**
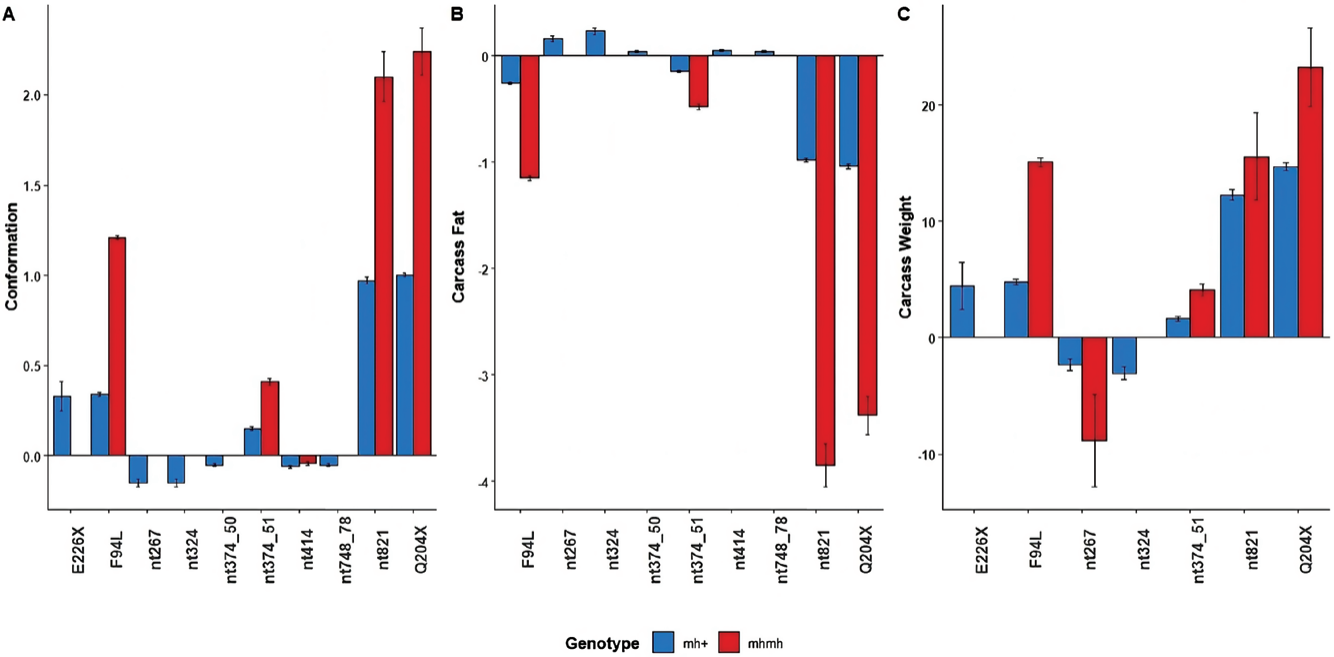
The effect of the mutant allele (mh) vs. the wild-type allele (+) for each variant on (A) carcass weight, (B) conformation, and (C) carcass fat (*P* < 0.05), where the error bars represent the standard error of the allele substitution effects. The genetic standard deviation for carcass weight, conformation, and carcass fat used in national genetic evaluations is 26.16 kg, 1.01 units, and 0.94 units, respectively.

**Figure 5.**
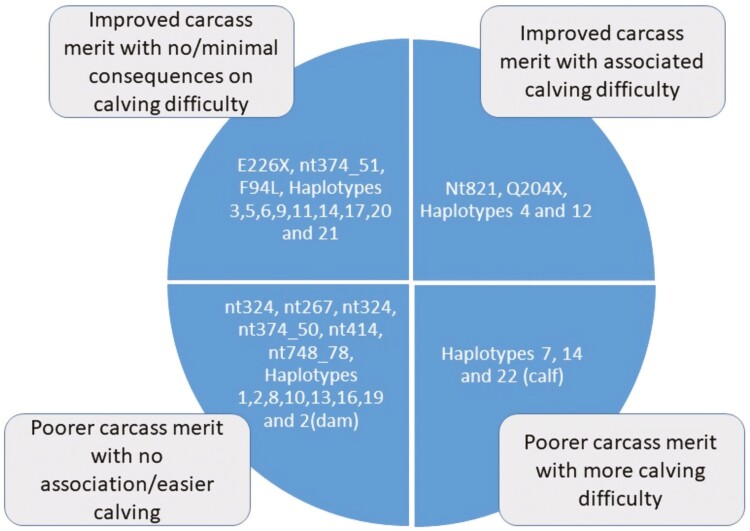
The association of myostatin mutant alleles and haplotypes with calving ease when carried by both the dam and the progeny, as well as their association with at least two of the three carcass traits (*P* < 0.05).

Mean (± SD) carcass weight, conformation score, and fat score in the sample population were 347.1 kg (±52.49), 8.36 units (±1.946), and 8.913 units (±1.971), respectively. Figure 4 illustrates how the average carcasses of animals carrying one or two mutant alleles per variant differ from animals carrying no mutant allele, and Figure 5 illustrates the variants and haplotypes that are favorable and unfavorable for calving difficulty and carcass traits. With the exception of nt748_78, nt414, and nt374_51, all other 7 segregating myostatin variants were associated with carcass weight. Variants associated with conformation were generally also associated with carcass fat; the mutant genotypes for all 10 segregating variants were associated with carcass conformation, and the mutant genotypes for all ­segregating variants other than E226X were associated with carcass fat; those mutant alleles that had a positive allele substitution effect on conformation had a negative allele substitution effect on fat. The F94L, Q204X, and nt821 variants ­generally had the largest observed associations with all three carcass traits (Figure 4). Animals with the mh/mh genotype for nt821 had carcasses that were, on average, 15.56 kg heavier, 2.1 units superior in conformation score, and 3.85 units leaner relative to animals with the +/+ nt821 genotype. Similarly, animals homozygous for the mutant Q204X allele had carcasses that were, on average, 23.2 kg heavier, 2.24 units superior in conformation score, and 3.38 units leaner relative to animals with homozygous wild-type +/+ Q204X genotype. The carcasses of animals with the homozygous mutant type genotype for the F94L variant were, on average, 15.06 kg heavier, 1.21 units higher conformation score, and were 1.15 units leaner relative to animals with the +/+ F94L genotype. When all the variants that had an association with the phenotype under investigation were fitted in the model together, nt374_51, nt267, and nt324 were no longer associated with carcass weight, and nt267 and nt748_78 were no longer associated with carcass conformation or fat (Table 8).

**Table 8.** The effect^1^ (standard error in parentheses) of carrying one or two copies (vs. no copy) for all the variants fitted concurrently in the multi-locus association for carcass conformation, fat, weight, and calving difficulty

		Conformation		Fat		Weight		Calf calving difficulty		Dam calving difficulty	
Variant	Genotype	*P*-value	Effect (SE)	*P*-value	Effect (SE)	*P*-value	Effect (SE)	*P*-value	Effect (SE)	*P*-value	Effect (SE)
E226X	mh+	<0.01	0.4(0.07)	0.80	-0.03(0.11)	0.01	5.47(2.00)	NA	NA	NA	NA
F94L	mh+	<0.01	0.54(0.01)	<0.01	-0.26(0.01)	<0.01	7.61(0.26)	0.02	0.01(0.01)	0.04	−0.02(0.01)
F94L	mhmh	<0.01	1.6(0.01)	<0.01	1.21(0.01)	<0.01	20.6(0.40)	<0.01	0.07(0.02)	0.57	−0.01(0.02)
nt267	mhmh	0.9	−0.02(0.14)	<0.01	0.16(0.03)	0.11	−6.2(3.87)	NA	NA	NA	NA
nt267	mh+	0.93	0(0.02)	0.43	0.17(0.22)	0.46	−0.36(0.49)	NA	NA	NA	NA
nt324	mh+	<0.01	0.1(0.02)	<0.01	0.23(0.03)	0.25	5.93(5.12)	0.13	−0.04(0.03)	NA	NA
nt324	mhmh	0.13	0.27(0.18)	0.69	0.11(0.28)	0.46	0.41(0.55)	0.13	0.23(0.15)	NA	NA
nt374_50	mh+	<0.01	0.13(0.03)	<0.01	0.04(0.01)	NA	NA	NA	NA	NA	NA
nt374_50	mhmh	<0.01	0.26(0.06)	0.51	0.01(0.02)	NA	NA	NA	NA	NA	NA
nt374_51	mhmh	<0.01	0.11(0.02)	<0.01	-0.15(0.01)	0.29	0.56(0.52)	0.92	0.01(0.03)	NA	NA
nt374_51	mh+	0.07	0.01(0.01)	<0.01	-0.48(0.03)	0.87	−0.03(0.21)	0.58	0.01(0.01)	NA	NA
nt414	mh+	<0.01	−0.16(0.03)	<0.01	0.05(0.01)	NA	NA	NA	NA	NA	NA
nt414	mhmh	<0.01	−0.35(0.06)	0.31	0.02(0.02)	NA	NA	NA	NA	NA	NA
nt748_78	mhmh	0.06	0.02(0.01)	<0.01	0.01(0.02)	NA	NA	NA	NA	NA	NA
nt748_78	mh+	0.71	0.01(0.02)	0.58	0.04(0.01)	NA	NA	NA	NA	NA	NA
nt821	mh+	<0.01	1.29(0.02)	<0.01	-0.98(0.02)	<0.01	16.56(0.48)	<0.01	0.42(0.11)	0.01	0.05(0.02)
nt821	mhmh	<0.01	2.62(0.13)	<0.01	-3.85(0.20)	<0.01	23.27(3.70)	<0.01	0.16(0.02)	<0.01	1.29(0.13)
Q204X	mh+	<0.01	1.27(0.01)	<0.01	-1.04(0.02)	<0.01	17.88(0.35)	<0.01	0.11(0.02)	0.03	−0.06(0.03)
Q204X	mhmh	<0.01	2.75(0.12)	<0.01	-3.38(0.18)	<0.01	29.02(3.35)	0.06	0.29(0.15)	NE	NE

^1^NA refers to whether the genotype effect was not significant or not present in the single locus association for the phenotype under investigation.

^2^NE refers to when no animals were present in this study with that genotype.

Of the 22 haplotypes, 18 were associated with carcass weight, conformation, or fat (*P* < 0.05) (Table 9). The haplotype containing just the nt748_78, nt374_50, nt414, and Q204X mutant alleles generally had the largest observed association with all three carcass traits; the carcasses of animals homozygous for this haplotype were, on average, 15.73 kg heavier, 1.90 units better in conformation score, and were 3.12 units leaner relative to animals with no copy of this haplotype.

**Table 9.** The number of copies of each haplotype carried by the animal that was associated with carcass weight, conformation, and fat, the mutant alleles contained in each haplotype, and the effect^1^ (standard error in parentheses) of carrying one or two copies (vs. no copy) of that haplotype on carcass weight, conformation, and fat

Haplotype	Mutant alleles	Number of copies	Weight	Conformation	Fat
			*P* < 0.05	*P* < 0.05	*P* < 0.05
1	None	1	−5.04(0.20)	−0.41(0.01)	0.43(0.01)
1	None	2	−7.81(0.33)	−0.58(0.01)	0.53(0.02)
2	nt414, nt374_50, nt748_78	1	−2.97(0.25)	−0.22(0.01)	0.16(0.01)
2	nt414, nt374_50, nt748_78	2	−5.51(0.50)	−0.34(0.02)	0.24(0.03)
3	F94L	1	1.35(0.23)	0.11(0.01)	−0.07(0.01)
3	F94L	2	8.37(0.37)	0.69(0.01)	−0.64(0.02)
4	nt821, nt374_51	1	4.05(0.66)	0.33(0.03)	−0.26(0.04)
4	nt821, nt374_51	2	9.56(4.20)	1.90(0.16)	−3.58(0.23)
5	F94L, nt374_51	2	3.66(0.87)	0.42(0.03)	−0.52(0.05)
5	F94L, nt374_51	1	0.72(0.28)	0.07(0.01)	−0.07(0.02)
6	nt374_51	2	NA	−0.27(0.08)	NA
7	nt748_78	2	−1.93(0.77)	−0.14(0.03)	0.09(0.04)
7	nt748_78	1	NA	−0.05(0.02)	0.08(0.03)
8	nt267	1	NA	−0.13(0.04)	0.12(0.05)
9	nt748_78, nt374_50, nt414, Q204X	1	9.56(0.43)	0.53(0.02)	−0.38(0.02)
9	nt748_78, nt374_50, nt414, Q204X	2	15.73(3.96)	1.9(0.15)	−3.12(0.22)
10	nt748_78, nt374_51nt374_50, nt414	1	NA	−0.17(0.04)	0.16(0.06)
11	nt748_78, nt267, nt374_50, nt414	1	15.52(0.64)	1.17(0.02)	−1.21(0.03)
12	F94L, nt374_51, nt821	1	11.01(5.20)	0.49(0.20)	NA
13	E226X, nt374_51	1	2.39(1.11)	NA	NA
13	E226X, nt374_51	2	NA	−0.12(0.04)	0.21(0.06)
14	nt374_50, nt414	1	15.69(0.59)	1.23(0.02)	−1.51(0.03)
15	F94L, nt374_50, nt414, Q204X	1	−2.67(0.29)	−0.25(0.01)	0.33(0.02)
15	F94L, nt374_50, nt414, Q204X	2	NA	NA	−1.20(0.52)
16	nt748_78,F94L, nt374_50, nt414	1	−3.28(1.00)	−0.2(0.04)	0.30(0.05)
16	nt748_78, F94L, nt374_50, nt414	2	NA	−0.51(0.25)	NA
17	nt324	1	3.44(1.72)	0.19(0.07)	NA
17	nt324	2	NA	2.31(1.08)	−5.01(1.54)
18	nt748_78, nt374_51, nt821	1	NA	NA	0.18(0.09)
19	F94L, nt324, nt374_50	1	−3.48(0.66)	−0.25(0.03)	0.23(0.04)
20	nt267, nt374_51	2	6.11(1.12)	0.48(0.04)	−0.48(0.06)
21	F94L, nt748_78	1	13.05(1.10)	1.06(0.04)	−1.36(0.06)
22	nt748_78,F94L,nt374_50,nt374_51,nt414,Q204X,E226X	1	−5.17(2.24)	−0.17(0.09)	0.28(0.12)

^1^NA refers to if the effect was not significantly different from zero.

## Discussion

One of the aims of the present study was to first determine the percent of animals carrying at least one mutant allele for the 21 known myostatin mutations in a large study population of 12 prominent *Bos taurus* dairy and beef breeds. Of the 10 segregating myostatin variants detected, only three demonstrated pleiotropy associated with calving difficulty and carcass traits; the nt821 and Q204X mutant alleles were both associated with both larger and more conformed carcasses but also a greater genetic predisposition to difficult calving; the intronic nt324 mutant allele, on the other hand, was associated with lighter and less conformed carcasses but also easier calving. The LD between these variants and all other segregating myostatin variants was ≤ 0.2 in the admixed population, highlighting that the estimated allele substitution effects of these three variants are independent of one another.

### Improved carcass merit with minimal consequences on calving difficulty

Calving difficulty and carcass merit are antagonistically genetically correlated in cattle (Berry et al., 2019), with previous studies reporting a moderate to strong genetic correlation between calving difficulty and both carcass weight (0.30 to 0.64) (Berry et al., 2019; Hosono et al., 2020) and carcass fat (0.42 to 0.54) (Eriksson et al., 2004; Hosono et al., 2020) with a weaker genetic correlation between calving difficulty and carcass conformation (0.1) (Berry et al., 2019). These antagonistic relationships suggest that selecting sires to minimize calving difficulty tends to, on average, produce smaller calves with inferior carcasses (Hosono et al., 2020; Twomey et al., 2020). That being said, the nonsense exonic E226X G to T substitution that results in a premature stop codon, the missense exonic F94L C to A substitution, and the silent intronic nt374_51 C to T substitution were all associated with an improved carcass merit substitution effect for carcass weight, with little to no expected consequence on calving difficulty (for the genotype of the calf or the cow). Therefore, despite the antagonistic correlation between calving difficulty and carcass traits, it is possible to identify animals carrying these mutation(s) that could potentially have good genetic merit for both calving difficulty and carcass merit. This may be particularly useful for maximizing the value of surplus calves from the dairy industry at slaughter without negatively impacting the cow’s reproductive performance, when both calving difficulty and carcass merit are a priority (Berry and Ring, 2020). The allele substitution effect of E226X, F94L, and nt374_51was not negligible, and represented from 0.1% to 1.4% of the genetic variance for carcass weight, 0.6% to 5% of the genetic variance for carcass conformation, and 0.6% to 6.6% of the genetic variance for carcass fat.

### Myostatin mutations associated with improved carcass merit and greater calving difficulty

The greatest pleiotropic effect evident in the present study was for the nt821 11 base pair deletion and for the Q204X C to T substitution, both of which resulted in a premature stop codon. The observed pleiotropy in the present study manifested itself as an association between the nt821 deletion and the Q204X mutant allele carried by the progeny with greater calving difficulty and leaner, heavier carcasses with better conformation, which is not surprising, given the moderate to strong genetic correlation between calving difficulty and carcass merit in cattle (Berry et al., 2019). Despite the advantage animals carrying the Q204X or nt821 mutation(s) have on carcass traits (Arthur, 1995; Casas et al., 1998), the disadvantage of the associated calving difficulty has resulted in selection against these mutations in many breeds (Taylor, 2017; Aiello et al., 2018), as was the case with Gascon (Phocas, 2009) and Marchigiana cattle (Ceccobelli et al., 2022).

A larger calf relative to a small pelvic area of the dam has been reported as the primary cause of dystocia (Naazie et al., 1989; Nugent et al., 1991). Dams carrying two copies of the nt821 or Q204X mutations have been reported to have a narrower pelvic opening (Arthur et al., 1988; Taylor, 2017) but also generate a bigger calf (Short et al., 2002) compared to dams without these mutations. While a larger calf may not cause calving difficulty for dams homozygous for the nt821 or Q204X wild-type variants (Figure 2), calving difficulty tends to be exacerbated in double-muscled dams carrying the nt821 or Q204X mutations due to their narrower pelvic opening (Taylor, 2017). This narrow pelvic opening of double-muscled dams is likely to be attributable to a relative reduction in the size of the pelvic girdle which is accompanied by more angular convergence of the iliac branches of the hipbones (Arthur et al., 1988; Taylor, 2017). A similar ­phenomenon was observed in the present study, in which calves and dams carrying one or two copies of the nt821 deletion and homozygous (albeit not significantly different from zero due to a paucity of numbers) and heterozygous calves carrying the Q204X mutation experienced the most calving difficulty out of all the segregating variants, relative to their homozygous wild-type contemporaries.

Many previous studies exploring associations between myostatin genotypes and calving difficulty have focused primarily on the myostatin genotype of the animal (Casas et al., 1999; Wiener et al., 2002). The study herein demonstrated that for many myostatin variants, the association between the calf’s genotype and calving difficulty actually differed depending on the genotype of the dam (Figure 2). This highlights that calving difficulty is a complex trait controlled by both genes affecting the ability of the calf to be born easily (direct genetic effects) and by genes affecting the ability of the cow to give birth without problems (maternal genetic effects) (Meijering, 1984). Therefore, by selectively mating a bull with two copies of the nt821 or Q204X mutation to a dam with a wide pelvic opening, the progeny should have improved carcass merit while minimizing associated risk of calving difficulty.

It has been suggested that the genotype of the calf is more influential than the genotype of the dam in determining the difficulty of calving (Wiener et al., 2002). If there was a substantial effect of the mutant maternal genotype, one might expect to detect it in the comparison of the calving difficulty between mh/+ dams and +/+ calves relative to +/+ dams and +/+ calves. Similarly, if there was a substantial effect of the mutant calf genotype, one might expect to see it in the comparison of the calving difficulty between +/+ dams and mh/+ calves relative to +/+ dams and +/+ calves. The effect of nt821 mh/+ dams and +/+ calves on calving difficulty was, however, not different from +/+ +/+ dam-calf combinations. In comparison, nt821 +/+ dams and mh/+ calves had, on average, 0.10 points higher calving difficulty than +/+ +/+ dam-calf combinations. This suggests that an nt821 mh/+ calf influences calving difficulty more than an nt821 mh/+ dam does. However, there may be more of a dam effect influencing calving difficulty or a combination of a dam and calf effect for nt821 mh/mh dams, given that double-muscled dams have a narrower pelvic width than mh/+ dams (Short et al., 2002).

### Allele and haplotype frequencies

The low-frequency mutant allele for some variants in some populations suggests the mutant allele called in those animals originated from either a de novo mutation, crossbreeding, or a genotyping error. Genotyping errors are known to impact allele and haplotype frequency estimates (Quade et al., 2005; Govindarajulu et al., 2006; Zhu et al., 2007), and are inevitable when genotyping a large number of animals (Kirk and Cardon, 2002). All animals included in the study herein were checked for Mendelian errors where possible. Of the 725 purebred animals that carried a mutant allele that was present in just 1% of their breed, the parentage of 257 animals were verified using the genotypes of their parent(s) and 144 animals were checked against the genotypes of their progeny; no discrepancies were ever detected. Hence, for these animals at least, the called genotype is likely to be the true genotype.

The eight most prevalent haplotypes, which are presumably the oldest haplotypes (Watterson and Guess, 1977; Dunner et al., 2003), generally carried only one or two mutant alleles each. The mutations present in the most prevalent haplotypes (excluding the wild-type haplotype) were possibly favored over generations due to selection for larger muscle mass, thereby proliferating in the breeds (Gautier et al., 2017). However, there are other possible explanations as to why these mutations were present in the most prevalent haplotypes, such as population segmentation followed by fixation of alleles. The less prevalent haplotypes identified in the present study generally carried several mutant alleles. They may have evolved from the older, more frequent haplotypes, possibly as a result of recombination events interrupting the LD phase flanking the original haplotype’s single mutant allele or as a result of a more recent mutation arising in the population.

While the nt821 deletion is almost fixed in the Belgian Blue population (Smith et al., 2000; Konovalova et al., 2021a), it has been introduced in other breeds at a low frequency (Gill et al., 2009; Konovalova et al., 2021b). Because the haplotype with the nt821 deletion and the nt374_51 mutation was detected at a low frequency in the Angus population but was common in the Belgian Blue population, it is likely that the nt821 deletion was introduced into the Angus population through crossbreeding prior to 2003; the oldest Angus animal in the present study with that haplotype was born in 2003. Given that Belgian Blues excel in genetic merit for carcass traits whereas Angus cattle are, on average, genetically easier calving (Berry and Ring, 2020), mating them together is likely to result in larger and more conformed carcasses with hopefully still an acceptable genetic predisposition to calving difficulty. A PCA of the purebred Angus and Belgian Blue carriers of the haplotype containing the nt821 and the nt374_51 mutations is shown in Supplementary Figure 2; this shows a distinct, non-overlapping cluster for both breeds.

The haplotype containing the nt821 deletion (with the F94L and nt374_51 mutations) that was found at a low frequency in the Aubrac and Limousin populations in this study was absent in the Belgian Blue population, and therefore, is possibly a consequence of a recent de novo mutation, which appeared in the Irish Aubrac and Limousin populations prior to 1998 and 2004, respectively. De novo mutations are very rare, with Holstein-Friesian cattle reported to have a baseline spontaneous de novo mutation rate of ~1.2 × 10^−8^ mutations per base pair, per generation (Coppieters et al., 2018). Haruna et al. (2020) suggested that the myostatin gene harbors recombination hotspots, which could potentially increase the likelihood of a myostatin de novo deletion occurring. Meiotic recombination, which is localized in recombination hotspots, not only increases genetic diversity through the formation of new haplotypes but is also strongly suspected to be mutagenic due to recurrent double-strand breaks (Arbeithuber et al., 2015). In particular, Lukaszewicz et al. (2021) recently demonstrated that double cutting within single double-strand breaks at recombination hotspots can initiate microdeletions in mice. Furthermore, an enrichment for structural variants and an elevated rate of rare alleles at recombination hotspots in humans has also been documented by Beyter et al. (2021).

While gene editing has been used to introduce the nt821 deletion into the genome of Wagyu cattle for the improvement of carcass merit (Tan et al., 2013), beneficial myostatin mutations can also be introduced to a population by crossbreeding and then selecting progeny with the desirable allele (Hansen, 2011). Exploiting crossbreeding for the introduction of myostatin mutations associated with larger and more conformed carcasses may be particularly useful for improving the carcass merit of numerically smaller, local breeds, given that the performance gap between high-yielding breeds with large populations vs. local breeds has increased in recent decades (Stock et al., 2022). Crossbreeding, in tandem with genotyping, can make alleles that were introduced at a low frequency become prominent within several generations (Amador et al., 2011). This means that genetic gain for the trait of interest could be achieved relatively quickly, as was realized when high-yielding Montbéliarde and Red Holstein alleles were introduced into the small German Vorderwald breed to increase milk yield (Hartwig et al., 2014).

## Conclusion

Only the nt821 and Q204X mutations were associated with both larger and more conformed carcasses, but also with a greater genetic predisposition to difficult calving. The association between the calf’s genotype and calving difficulty differed depending on the genotype of the dam. Therefore, it is possible to produce progeny with improved carcass merit from a bull with two copies of the nt821 or Q204X mutation while minimizing the associated risk of calving difficulty, depending on the genotype of the dam. Despite the antagonistic relationship between calving difficulty and carcass traits, the nt374_51, F94L, and E226X mutations were all associated with improved carcass merit while having minimal consequences on calving difficulty. Therefore, it is possible to identify bulls carrying these mutation(s) that could potentially have good genetic merit for both calving difficulty and carcass merit.

## Supplementary Material

skad371_suppl_Supplementary_Figures_S1

skad371_suppl_Supplementary_Figures_S2

## Abbreviations

EUROP: European Union beef carcass classification system
GDF8: myostatin
ICBF: Irish Cattle Breeding Federation
LD: linkage disequilibrium
PCA: principal component analysis
SNP: single nucleotide polymorphism

## References

[CIT0001] Aiello, D., K. Patel, and E. Lasagna. 2018. The myostatin gene: an overview of mechanisms of action and its relevance to livestock animals. Anim. Genet. 49:505–519. doi: 10.1111/age.12696 30125951

[CIT0002] Alexander, D. H., J. Novembre, and K. Lange. 2009. Fast model-based estimation of ancestry in unrelated individuals. Genome Res. 19:1655–1664. doi: 10.1101/gr.094052.109 19648217 PMC2752134

[CIT0003] Amador, C., M. Toro, and J. Fernández. 2011. Removing exogenous information using pedigree data. Conserv. Genet. 12:1565–1573. doi: 10.1007/s10592-011-0255-4

[CIT0004] Arbeithuber, B., A. J. Betancourt, T. Ebner, and I. Tiemann-Boege. 2015. Crossovers are associated with mutation and biased gene conversion at recombination hotspots. Proc. Natl. Acad. Sci. U.S.A. 112:2109–2114. doi: 10.1073/pnas.1416622112 25646453 PMC4343121

[CIT0005] Arthur, P. 1995. Double muscling in cattle: a review. Aust. J. Agric. Res. 46:1493. doi: 10.1071/ar9951493

[CIT0006] Arthur, P. F., M. Makarechian, and M. A. Price. 1988. Incidence of dystocia and perinatal calf mortality resulting from reciprocal crossing of double-muscled and normal cattle. Can. Vet. J. 29:163–167. https://www.ncbi.nlm.nih.gov/pmc/articles/PMC1680682/17422971 PMC1680682

[CIT0007] Barrett, J. C., B. Fry, J. Maller, and M. J. Daly. 2004. Haploview: analysis and visualization of LD and haplotype maps. Bioinformatics 21:263–265. doi: 10.1093/bioinformatics/bth457 15297300

[CIT0008] Berry, D. P., and R. D. Evans. 2014. Genetics of reproductive performance in seasonal calving beef cows and its association with performance traits. J. Anim. Sci. 92:1412–1422. doi: 10.2527/jas.2013-6723 24496848

[CIT0009] Berry, D. P., and S. C. Ring. 2020. Observed progeny performance validates the benefit of mating genetically elite beef sires to dairy females. J. Dairy Sci. 103:2523–2533. doi: 10.3168/jds.2019-17431 31928752

[CIT0010] Berry, D. P., P. R. Amer, R. D. Evans, T. Byrne, A. R. Cromie, and F. Hely. 2019. A breeding index to rank beef bulls for use on dairy females to maximize profit. J. Dairy Sci. 102:10056–10072. doi: 10.3168/jds.2019-16912 31495621

[CIT0011] Beyter, D., H. Ingimundardottir, A. Oddsson, H. P. Eggertsson, E. Bjornsson, H. Jonsson, B. A. Atlason, S. Kristmundsdottir, S. Mehringer, M. T. Hardarson, et al. 2021. Long-read sequencing of 3,622 Icelanders provides insight into the role of structural variants in human diseases and other traits. Nat. Genet. 53:779–786. doi: 10.1038/s41588-021-00865-4 33972781

[CIT0012] Blott, S. C., J. L. Williams, and C. S. Haley. 1998. Genetic variation within the Hereford breed of cattle. Anim. Genet. 29:202–211. doi: 10.1046/j.1365-2052.1998.00326.x 9720179

[CIT0013] Boman, I., G. Klemetsdal, T. Blichfeldt, O. Nafstad, and D. Våge. 2009. A frameshift mutation in the coding region of the myostatin gene (MSTN) affects carcass conformation and fatness in Norwegian White Sheep (Ovis aries). Anim. Genet. 40:418–422. doi: 10.1111/j.1365-2052.2009.01855.x 19392824

[CIT0014] Casas, E., J. W. Keele, S. D. Shackelford, M. Koohmaraie, T. S. Sonstegard, T. P. Smith, S. M. Kappes, and R. T. Stone. 1998. Association of the muscle hypertrophy locus with carcass traits in beef cattle. J. Anim. Sci. 76:468–473. doi: 10.2527/1998.762468x 9498354

[CIT0015] Casas, E., J. W. Keele, S. C. Fahrenkrug, T. P. Smith, L. V. Cundiff, and R. T. Stone. 1999. Quantitative analysis of birth, weaning, and yearling weights and calving difficulty in Piedmontese crossbreds segregating an inactive myostatin allele. J. Anim. Sci. 77:1686–1692. doi: 10.2527/1999.7771686x 10438013

[CIT0016] Ceccobelli, S., F. Perini, M. F. Trombetta, S. Tavoletti, E. Lasagna, and M. Pasquini. 2022. Effect of myostatin gene mutation on slaughtering performance and meat quality in marchigiana bulls. Animals (Basel). 12:518. doi: 10.3390/ani12040518 35203227 PMC8868461

[CIT0017] Chung, Y., K. Chung, P. Dinh, I. Choi, and S. Lee. 2022. Finding potentially causal genetic factors for meat tenderness using eQTL study in Hanwoo cattle WCGALP 2022. Rotterdam: WCGALP.

[CIT0018] Clop, A., F. Marcq, H. Takeda, D. Pirottin, X. Tordoir, B. Bibé, J. Bouix, F. Caiment, J. -M. Elsen, F. Eychenne, et al. 2006. A mutation creating a potential illegitimate microRNA target site in the myostatin gene affects muscularity in sheep. Nat. Genet. 38:813–818. doi: 10.1038/ng1810 16751773

[CIT0019] Coppieters, W., C. Charlier, M. Georges, C. Harland, and E. Mullaart. 2018. Rate of de novo mutation in dairy cattle and potential impact of reproductive technologies. Auckland: WCGALP.

[CIT0020] Crump, R. E., N. R. Wray, R. Thompson, and G. Simm. 1997. Assigning pedigree beef performance records to contemporary groups taking account of within-herd calving patterns. Anim. Sci. 65:193–198. doi: 10.1017/s1357729800016490

[CIT0021] Dunner, S., M. E. Miranda, Y. Amigues, J. Cañón, M. Georges, R. Hanset, J. Williams, and F. Ménissier. 2003. Haplotype diversity of the myostatin gene among beef cattlebreeds. Genet. Sel. Evol. 35:103. doi: 10.1186/1297-9686-35-1-103 12605853 PMC2732685

[CIT0022] Elzaki, S., P. Korkuc, D. Arends, M. Reissmann, and G. A. Brockmann 2021. Effects of DGAT1 on milk performance in Sudanese Butana × Holstein crossbred cattle. *Trop. Anim. Health. Prod*. 54(2):142. doi: 10.1007/s11250-022-03141-7 PMC8948139 35332362

[CIT0023] Englishby, T. M., G. Banos, K. L. Moore, M. P. Coffey, R. D. Evans, and D. P. Berry. 2016. Genetic analysis of carcass traits in beef cattle using random regression models1. J. Anim. Sci. 94:1354–1364. doi: 10.2527/jas.2015-0246 27135995

[CIT0024] Eriksson, S., A. Näsholm, K. Johansson, and J. Philipsson. 2004. Genetic relationships between calving and carcass traits for Charolais and Hereford cattle in Sweden. J. Anim. Sci. 82:2269–2276. doi: 10.2527/2004.8282269x 15318724

[CIT0025] Gautier, M., A. Klassmann, and R. Vitalis. 2017. <scp>rehh</scp>20: a reimplementation of the R package<scp>rehh</scp>to detect positive selection from haplotype structure. Mol. Ecol. Resour. 17:78–90. doi: 10.1111/1755-0998.12634 27863062

[CIT0026] Gill, J. L., S. C. Bishop, C. McCorquodale, J. L. Williams, and P. Wiener. 2009. Associations between the 11-bp deletion in the *myostatin* gene and carcass quality in Angus-sired cattle. Anim. Genet. 40:97–100. doi: 10.1111/j.1365-2052.2008.01790.x 18822096

[CIT0027] Gilmour, A. R., B. J. Gogel, Cullis., B. R., S. J. Welham, and R. Thompson. 2015. ASReml User Guide - Release 4.1. Structural Specification: VSN International Ltd, Hemel Hempstead, HP1 1ES, U.

[CIT0028] Govindarajulu, U. S., D. Spiegelman, K. L. Miller, and P. Kraft. 2006. Quantifying bias due to allele misclassification in case-control studies of haplotypes. Genet. Epidemiol. 30:590–601. doi: 10.1002/gepi.20170 16830341

[CIT0029] Grisart, B., W. Coppieters, F. Farnir, L. Karim, C. Ford, P. Berzi, N. Cambisano, M. Mni, S. Reid, P. Simon, et al. 2002. Positional candidate cloning of a QTL in dairy cattle: identification of a missense mutation in the bovine DGAT1 gene with major effect on milk yield and composition. Genome Res. 12:222–231. doi: 10.1101/gr.224202 11827942

[CIT0030] Grisolia, A., G. D’Angelo, L. Porto-Neto, F. Siqueira, and J. F. Garcia. 2009. Myostatin (GDF8) single nucleotide polymorphisms in Nellore cattle. Genet. Mol. Res. 8:822–830. doi: 10.4238/vol8-3gmr548 19731204

[CIT0031] Grobet, L., L. J. Martin, D. Poncelet, D. Pirottin, B. Brouwers, J. Riquet, A. Schoeberlein, S. Dunner, F. Ménissier, J. Massabanda, et al. 1997. A deletion in the bovine myostatin gene causes the double-muscled phenotype in cattle. Nat. Genet. 17:71–74. doi: 10.1038/ng0997-71 9288100

[CIT0032] Hansen, P. J. 2011. 4.41 - heat stress and climate change. In: M. Moo-Young, editor, Comprehensive Biotechnology (Third Edition). Pergamon: Oxford; 489–497.

[CIT0033] Hartwig, S., R. Wellmann, H. Hamann, and J. Bennewitz. 2014. The contribution of migrant breeds to the genetic gain of beef traits of German Vorderwald and Hinterwald cattle. J. Anim. Breed. Genet. 131:496–503. doi: 10.1111/jbg.12099 24965852

[CIT0034] Haruna, I. L., U. J. Ekegbu, F. Ullah, H. Amirpour-Najafabadi, H. Zhou, and J. G. H. Hickford. 2020. Genetic variations and haplotypic diversity in the Myostatin gene of New Zealand cattle breeds. Gene. 740:144400. doi: 10.1016/j.gene.2020.144400 31987910

[CIT0035] Hosono, M., H. Oyama, and K. Inoue. 2020. Genetic relationships of calving difficulty with birth measurements and carcass traits in Japanese Black cattle. Anim. Sci. J. 91:e13491. doi: 10.1111/asj.13491 33337571

[CIT0036] Kamalludin, M. H., A. Garcia-Guerra, M. C. Wiltbank, and B. W. Kirkpatrick. 2017. Trio, a novel high fecundity allele: I Transcriptome analysis of granulosa cells from carriers and noncarriers of a major gene for bovine ovulation rate†. Biol. Reprod. 98:323–334. doi: 10.1093/biolre/iox133 29088317

[CIT0037] Kehrli, M. E., Jr, F. C. Schmalstieg, D. C. Anderson, M. J. Van der Maaten, B. J. Hughes, M. R. Ackermann, C. L. Wilhelmsen, G. B. Brown, M. G. Stevens, et al. 1990. Molecular definition of the bovine granulocytopathy syndrome: identification of deficiency of the Mac-1 (CD11b/CD18) glycoprotein. Am. J. Vet. Res. 51:1826–1836. https://pubmed.ncbi.nlm.nih.gov/1978618/1978618

[CIT0038] Kenny, D., C. P. Murphy, R. D. Sleator, M. M. Judge, R. D. Evans, and D. P. Berry. 2020. Animal-level factors associated with the achievement of desirable specifications in Irish beef carcasses graded using the EUROP classification system. J. Anim. Sci. 98:skaa191. doi: 10.1093/jas/skaa191 32516387 PMC7333216

[CIT0039] Khasanaha, H., A. Gunawanb, R. Priyantob, M. F. Ulumc, and Jakariab. 2016. Polymorphism of myostatin (MSTN) promotergene and its association with growth and muscling traits in bali cattle. *Media Peternakan*. 39(2):95–103. doi: 10.5398/medpet.2016.39.2.95

[CIT0040] Kirk, K. M., and L. R. Cardon. 2002. The impact of genotyping error on haplotype reconstruction and frequency estimation. Eur. J. Hum. Genet. 10:616–622. doi: 10.1038/sj.ejhg.5200855 12357332

[CIT0041] Konovalova, E., O. Romanenkova, O. Kostyunina, and E. Gladyr. 2021a. The molecular bases study of the inherited diseases for the health mintenance of the beef cattle. Genes. 12:678. doi: 10.3390/genes12050678 33946496 PMC8147127

[CIT0042] Konovalova, E., O. Romanenkova, A. Zimina, V. Volkova, and A. Sermyagin. 2021b. Genetic variations and haplotypic diversity in the myostatin gene of different cattle breeds in Russia. Animals (Basel). 11:2810. doi:10.3390/ani11102810 34679835 PMC8532888

[CIT0043] Loh, P. R., P. Danecek, P. F. Palamara, C. Fuchsberger, R. Y. A, F. H. K, S. Schoenherr, L. Forer, S. McCarthy, G. R. Abecasis, et al. 2016. Reference-based phasing using the Haplotype Reference Consortium panel. Nat. Genet. 48:1443–1448. doi: 10.1038/ng.3679 27694958 PMC5096458

[CIT0044] Lukaszewicz, A., J. Lange, S. Keeney, and M. Jasin. 2021. De novo deletions and duplications at recombination hotspots in mouse germlines. Cell 184:5970–5984.e18.e5918. doi: 10.1016/j.cell.2021.10.025 34793701 PMC8616837

[CIT0045] McPherron, A. C., and S. -J. Lee. 1997. Double muscling in cattle due to mutations in the myostatin gene. Proc. Natl. Acad. Sci. U.S.A. 94:12457–12461. doi: 10.1073/pnas.94.23.12457 9356471 PMC24998

[CIT0046] Meijering, A. 1984. Dystocia and stillbirth in cattle — a review of causes, relations and implications. Livest. Prod. Sci. 11:143–177. doi: 10.1016/0301-6226(84)90057-5

[CIT0047] Ménissier, F. 1982. Present state of knowledge about the genetic determination of muscular hypertrophy or the double muscled trait in cattle. In: K. Ménissier, and M. Nijhoff, editors. *Current Topics in Veterinary Medicine and Animal Science, vol. 16: Muscle hypertrophy of genetic origin and its use to improve beef production*; pp 387–428.

[CIT0048] Meuwissen, T. H. E., and M. E. Goddard. 2022. On the advantage of identifying causal genetic variants for genomic prediction WCGALP 2022, Rotterdam.

[CIT0049] Meyers, S. N., T. G. McDaneld, S. L. Swist, B. M. Marron, D. J. Steffen, D. O’Toole, J. R. O’Connell, J. E. Beever, T. S. Sonstegard, and T. P. Smith. 2010. A deletion mutation in bovine SLC4A2 is associated with osteopetrosis in Red Angus cattle. BMC Genomics. 11:337. doi: 10.1186/1471-2164-11-337 20507629 PMC2891616

[CIT0050] Naazie, A., M. M. Makarechian, and R. T. Berg. 1989. Factors influencing calving difficulty in beef heifers. J. Anim. Sci. 67:3243–3249. doi: 10.2527/jas1989.67123243x 2613572

[CIT0051] Nugent, R. A., D. R. Notter and W. E. Beal. 1991. Body measurements of newborn calves and relationship of calf shape to sire breeding values for birth weight and calving ease. J. Anim. Sci. 69:2413–2421. doi: 10.2527/1991.6962413x 1885359

[CIT0052] Pabiou, T., W. Fikse, P. Amer, A. Cromie, A. Näsholm, and D. Berry. 2012. Genetic relationships between carcass cut weights predicted from video image analysis and other performance traits in cattle. Animal. 6:1389–1397. doi: 10.1017/s1751731112000705 22717237

[CIT0053] Phocas, F. 2009. Genetic analysis of breeding traits in a Charolais cattle population segregating an inactive myostatin allele. J. Anim. Sci. 87:1865–1871. doi: 10.2527/jas.2008-1426 19213706

[CIT0054] Qian, L., M. Tang, J. Yang, Q. Wang, C. Cai, S. Jiang, H. Li, K. Jiang, P. Gao, D. Ma, et al. 2015. Targeted mutations in myostatin by zinc-finger nucleases result in double-muscled phenotype in Meishan pigs. Sci. Rep. 5:14435. doi: 10.1038/srep14435 26400270 PMC4585837

[CIT0055] Quade, S. R., R. C. Elston, and K. A. Goddard. 2005. Estimating haplotype frequencies in pooled DNA samples when there is genotyping error. BMC Genet. 6:25. doi: 10.1186/1471-2156-6-25 15943883 PMC1156884

[CIT0056] Ryan, C. A., D. P. Berry, A. O’Brien, T. Pabiou, and D. C. Purfield. 2023. Evaluating the use of statistical and machine learning methods for estimating breed composition of purebred and crossbred animals in thirteen cattle breeds using genomic information. Front. Genet. 14:1120312. doi: 10.3389/fgene.2023.1120312 37274789 PMC10237237

[CIT0057] Schmitz, F., R. W. Everett, and R. L. Quaas. 1991. Herd-year-season clustering. J. Dairy Sci. 74:629–636. doi: 10.3168/jds.s0022-0302(91)78210-6

[CIT0058] Schuelke, M., K. R. Wagner, L. E. Stolz, C. Hübner, T. Riebel, W. Kömen, T. Braun, J. F. Tobin, and S. -J. Lee. 2004. Myostatin mutation associated with gross muscle hypertrophy in a child. N Engl. J. Med. 350:2682–2688. doi: 10.1056/NEJMoa040933 15215484

[CIT0059] Short, R. E., M. D. MacNeil, M. D. Grosz, D. E. Gerrard, and E. E. Grings. 2002. Pleiotropic effects in Hereford, Limousin, and Piedmontese F2 crossbred calves of genes controlling muscularity including the Piedmontese myostatin allele. J. Anim. Sci. 80:1–11. doi: 10.2527/2002.8011 11831504

[CIT0060] Smith, J. A., A. M. Lewis, P. Wiener, and J. L. Williams. 2000. Genetic variation in the bovine myostatin gene in UK beef cattle: allele frequencies and haplotype analysis in the South Devon. Anim. Genet. 31:306–309. doi: 10.1046/j.1365-2052.2000.00521.x 11105210

[CIT0061] Stock, J., H. Esfandyari, R. Wellmann, D. Hinrichs, and J. Bennewitz. 2022. Genomic rotational crossbreeding with advanced optimum contribution selection methods applied to simulated German ­Angler and German Holstein dairy cattle populations. J. Anim. Breed. Genet. 140:121–131. doi: 10.1111/jbg.12750 36449261

[CIT0062] Tan, W., D. F. Carlson, C. A. Lancto, J. R. Garbe, D. A. Webster, P. B. Hackett, and S. C. Fahrenkrug. 2013. Efficient nonmeiotic allele introgression in livestock using custom endonucleases. Proc. Natl. Acad. Sci. U.S.A. 110:16526–16531. doi: 10.1073/pnas.1310478110 24014591 PMC3799378

[CIT0063] Taylor, M. L. 2017. The relationship between the myostatin gene and calving ease in beef cattle: a review of published research literature. Agricultural Business Research Institute, Armidale NSW 2351: BreedPlan.

[CIT0064] Teng, J., S. Huang, Z. Chen, N. Gao, S. Ye, S. Diao, X. Ding, X. Yuan, H. Zhang, J. Li, et al. 2020. Optimizing genomic prediction model given causal genes in a dairy cattle population. J. Dairy Sci. 103:10299–10310. doi: 10.3168/jds.2020-18233 32952023

[CIT0065] Twomey, A. J., S. C. Ring, N. McHugh, and D. P. Berry. 2020. Carcass and efficiency metrics of beef cattle differ by whether the calf was born in a dairy or a beef herd. J. Anim. Sci. 98:skaa321. doi: 10.1093/jas/skaa321 33011776 PMC7751157

[CIT0066] VanRaden, P. M., and A. H. Sanders. 2003. Economic merit of crossbred and purebred US dairy cattle. J. Dairy Sci. 86:1036–1044. doi: 10.3168/jds.S0022-0302(03)73687-X 12703641

[CIT0067] Watterson, G. A., and H. A. Guess. 1977. Is the most frequent allele the oldest? Theor. Popul. Biol. 11:141–160. doi: 10.1016/0040-5809(77)90023-5 867285

[CIT0068] Wiener, P., J. A. Smith, A. M. Lewis, J. A. Woolliams, and J. L. Williams. 2002. Muscle-related traits in cattle: the role of the myostatin gene in the South Devon breed. Genet. Sel. Evol. 34:221–232. doi: 10.1186/1297-9686-34-2-221 12081809 PMC2705429

[CIT0069] Yoneda, K., Y. Moritomo, M. Takami, S. Hirata, Y. Kikukawa, and T. Kunieda. 1999. Localization of a locus responsible for the bovine chondrodysplastic dwarfism (bcd) on chromosome 6. Mamm. Genome. 10:597–600. doi: 10.1007/s003359901052 10341093

[CIT0070] Zhu, W. S., W. K. Fung, and J. Guo. 2007. Incorporating genotyping uncertainty in haplotype frequency estimation in pedigree studies. Hum. Hered. 64:172–181. doi: 10.1159/000102990 17536211

