# Supplementary figures and images for: Associations between polymorphisms in the myostatin gene with calving difficulty and carcass merit in cattle
Source: J Anim Sci. 2023 Nov 3;101:skad371. doi: 10.1093/jas/skad371 (PMC10684047; doi:10.1093/jas/skad371)

**Supplementary Figure 1**

| **AA**  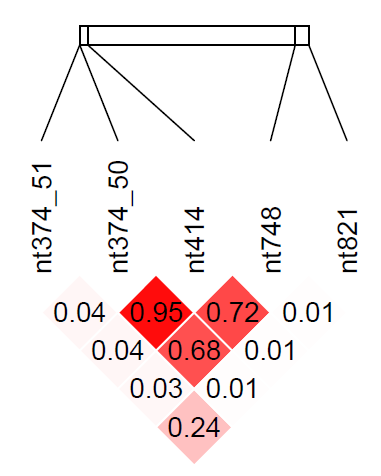 | **AU**  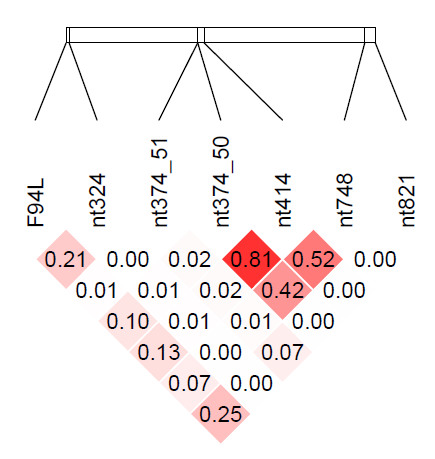 |
| --- | --- |
| **BA**  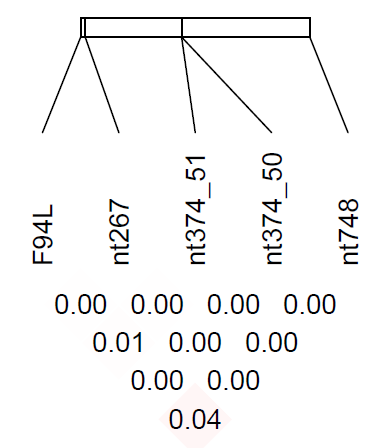 | **BB**  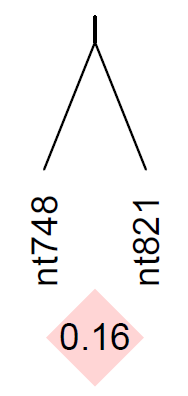 |
| **CH**  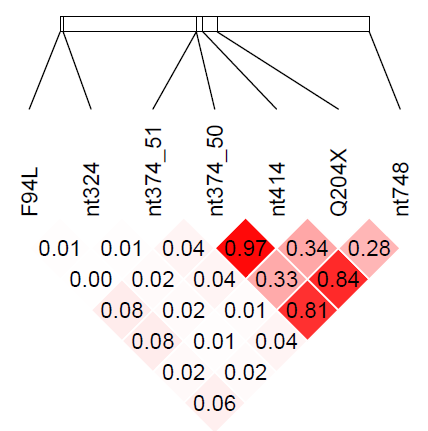 | **FR** 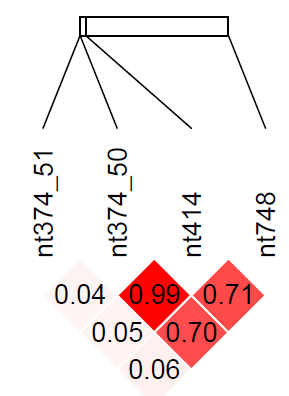 |
| **HE**  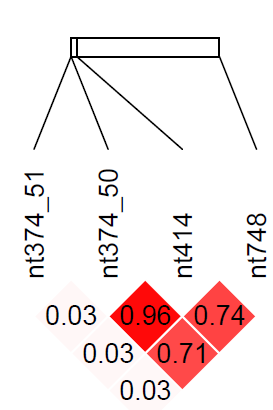 | **HO**  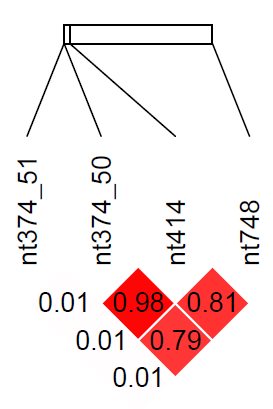 |
| **LM** 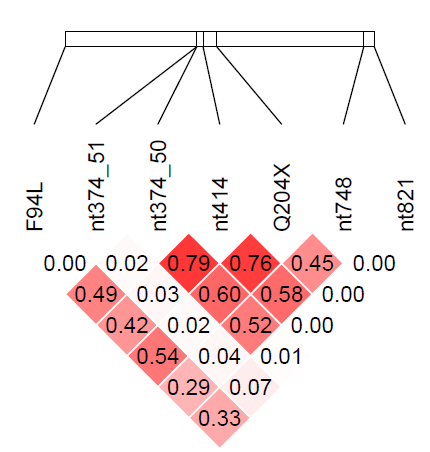 | **SA**  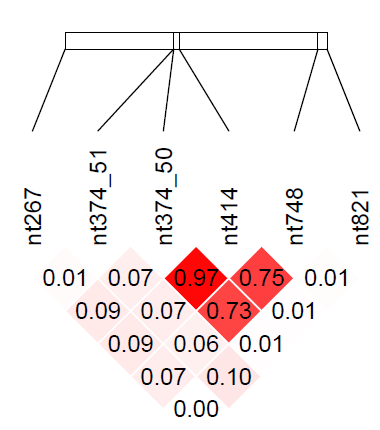 |
| **SH**  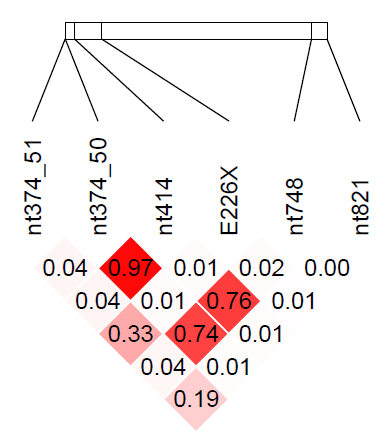 | 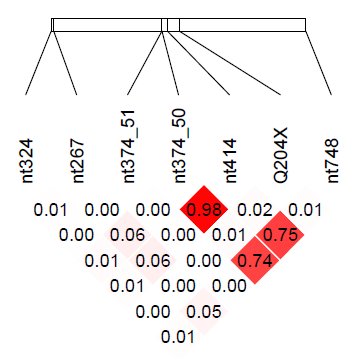**SI** |

Supplement: skad371_suppl_Supplementary_Figures_S1 [file skad371_suppl_supplementary_figures_s1.docx]

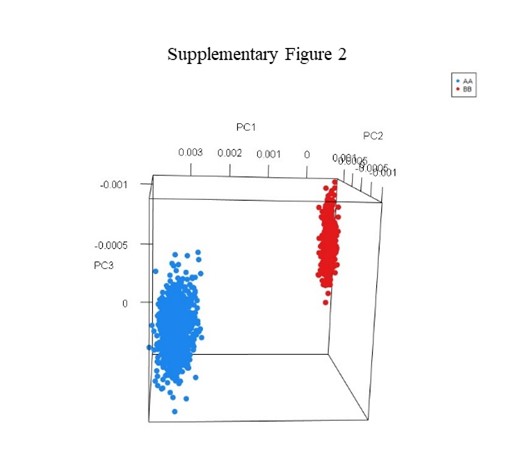

Supplement: skad371_suppl_Supplementary_Figures_S2 [file skad371_suppl_supplementary_figures_s2.jpeg]
